# Strain Distribution and Drug Susceptibility of Invasive Fungal Infection in Clinical Patients With Systemic Internal Diseases

**DOI:** 10.3389/fbioe.2020.625024

**Published:** 2021-02-11

**Authors:** Xuehua Zeng, Mengran Peng, Guirong Liu, Yongqing Huang, Tingting Zhang, Jing Wen, Wei Lai, Yue Zheng

**Affiliations:** ^1^Clinical Laboratory, The Third Affiliated Hospital of Sun Yat-sen University–Yuedong Hospital, Meizhou, China; ^2^Department of Dermatology, The Third Affiliated Hospital of Sun Yat-sen University, Guangzhou, China; ^3^Department of Dermatology, The Third Affiliated Hospital of Sun Yat-sen University–Yuedong Hospital, Meizhou, China; ^4^Department of Traditional Chinese Medicine, The Third Affiliated Hospital of Sun Yat-sen University–Yuedong Hospital, Meizhou, China

**Keywords:** invasive fungal infection, epidemiology, drug susceptibility, drug resistance, systemic internal diseases

## Abstract

**Background:**

Patients with systemic internal diseases present high risks for invasive fungal infections, which results in increased morbidity and mortality. Identification of high-risk departments and susceptibility systems could help to reduce the infective rate clinically. Correct selection of sensitive anti-fungal drugs not only could improve the cure rate but also could reduce the adverse reactions and complications caused by long-term antifungal drug treatment, which can be especially important in patients with serious systemic diseases. Therefore, the distribution changes of invasive fungal strains in patients with systemic internal diseases and the choice of antifungal drugs in clinical practice should be updated.

**Objective:**

This work aimed to investigate the incidence, strain distributions, and drug susceptibility of invasive fungal strains isolated from patients with systemic internal diseases.

**Methods:**

Samples were collected from 9,430 patients who were diagnosed with internal diseases in our hospital from January to December 2018. We then cultured and identified the fungal strains using API 20C AUX. We performed drug sensitivity analysis *via* the ATB Fungus-3 fungal susceptibility strip. Resistance was defined using the revised Clinical Laboratory Standardization Committee of United States breakpoints/epidemiological cutoff values to assign susceptibility or wild-type status to systemic antifungal agents.

**Results:**

A total of 179 patients (49 female, 130 male) with fungal infection were included. The high-incidence departments were determined to be the respiratory department (34.64%), intensive care unit (ICU; 21.79%), and hepatology department (9.50%). The susceptible systems for infection were the respiratory tract (sputum, 68.72%, 123/179; secretion retained in the tracheal catheter, 3.35%, 6/179), urinary tract (urine, 9.50%, 17/179), and gastrointestinal tract (feces, 9.50%, 17/179). The major pathogens were *Candida* (90.50%), *Aspergillus* (8.93%), and *Cryptococcus neoformans* (0.56%). The infective candida subgroups were *Candida albicans* (70.95%), *Candida krusei* (6.15%), *Candida glabrata* (5.59%), *Candida parapsilosis* (3.91%), and *Candida tropicalis* (3.91%). The susceptibility of non-*Aspergillus* fungi for amphotericin B was 100.0%. The susceptibility rates of 5-fluorocytocine (5-FC) and voriconazole were 72.73 and 81.82%, respectively, for *C. krusei*, 98.43 and 100% for *C. albicans*, and 100% for both drugs for *C. glabrata*, *C. parapsilosis*, and *C. tropicalis*. The susceptibility rates of fluconazole and itraconazole were 0 and 54.55%, respectively, for *C. krusei*, 20 and 20% for *C. glabrata*, and 57.14 and 57.14% for *C. tropicalis*. The resistance rate of *C. tropicalis* for both fluconazole and itraconazole was 41.43%.

**Conclusion:**

Patients in the respiratory department, ICU, and hepatology department presented high rates of invasive fungal infections and should include special attention during clinical treatment. The respiratory tract, urinary tract, and gastrointestinal tract were the susceptible systems. *Candida*, especially *C. albicans*, was the main pathogen. From the perspective of drug sensitivity, amphotericin B should be given priority in treating the non-*Aspergillus* fungi infection in patients with systemic internal diseases, while the susceptibility of invasive fungal strains to azoles was variant. These data might provide clinical evidence for the prevention and treatment of invasive fungal infection in patients with systemic internal diseases.

## Introduction

Patients with systemic internal diseases present an increasing risk of invasive fungal infection. In recent years, the extensive use of broad-spectrum antibiotics, hormones, immunosuppressants, and other drugs used for the treatment of patients with internal diseases has given rise to an increase in the rate of invasive fungal infection. Furthermore, the development and extension of new technologies for organ transplantation and other procedures might also induce infections from conditional pathogens, especially fungal infections, as well as increase the likelihood to confer drug resistance changes (Bonduel et al., 2001). Identification of high-risk departments and susceptibility systems which are prone to suffer from invasive fungal infection could help reduce the clinical infective rate.

The drug sensitivity and resistance of invasive fungi to the frequent use of fluconazole and itraconazole in clinical treatment are changing. Some of the mechanisms for antifungal drug resistance include drug absorption and drug accumulation, decreased affinity of the drug to its target, alteration of metabolic pathways to disturb cellular drug concentrations, and biofilm formation (Ramana et al., 2013). Factors related to a patient’s clinical situation and present co-morbidities, local epidemiology data, and purpose of treatment (prophylactic, pre-emptive, empiric, or definitive) should be taken into account when choosing the appropriate antifungal agents (Paramythiotou et al., 2014).

For the distribution of invasive fungi and drug sensitivity are varied, clinicians should select appropriate treatment schemes according to the patient’s condition and the drug sensitivity results. Clinical awareness and knowledge of local epidemiology and pharmaceutical considerations could also help to achieve early diagnosis and treatment. Correct selection of sensitive anti-fungal drugs could not only improve the cure rate but also could reduce adverse reactions and complications caused by long-term antifungal drug treatment, especially in patients with serious systemic diseases. Therefore, knowledge of the susceptibility and resistance of invasive fungi strains to antifungal agents should be updated, especially in patients with systemic internal diseases who have received therapeutic drugs.

In this study, we investigated the incidence, strain distributions, and drug susceptibility of invasive fungal strains isolated from patients with systemic internal diseases in the southern area of China.

## Materials and Methods

### Fungal Strain Collection

Both outpatient and inpatient samples, including sputum, urine, feces, blood, bile, cerebrospinal fluid, and secretion retained in the tracheal catheter, were collected from January to December 2018 at the Third Affiliated Hospital of Sun Yat-sen University–Yuedong Hospital. The samples were collected from 9,430 patients who were diagnosed with internal diseases, and a total of 179 strains of invasive fungi were isolated.

### Ethics

This work was an antifungal susceptibility surveillance study, and no human rights issues were involved. We obtained these strains in anonymized and de-identified forms.

### Instruments and Reagents

The *Candida* chromogenic culture medium was purchased from Jiangmen Kailin Trading Co., Ltd. We used the API 20C AUX identification instrument and ATB Fungus-3 fungal susceptibility strip by the French biological company Merieux.

### Culture and Identification

The cultures were incubated in Sabouraud media and maintained at 35°C for 24–48 h in order to grow into a yeast-like colony. The single colony was then inoculated into *Candida* chromogenic culture medium and identified with an API 20C-AUX system.

### Susceptibility Testing

We carried out the susceptibility test using the ATB Fungus 3 fungal susceptibility strip. Testing of the drug sensitivity of the minimum inhibitory concentration was obtained by utilizing ATB instrument interpretation. The strains were classified as sensitive, intermediate, or drug-resistant according to the Clinical Laboratory Standardization Committee of United States standard and ATB Fungus 3 product specification. The instructions were observed in detail, and the results were read with the naked eye if necessary. The quality control strain used was *Candida albicans*, ATCC 90028.

### Statistical Analysis

Data processing and statistical analysis were performed using SPSS software (version 16.0, Inc., Chicago, IL, United States). Count data variables were expressed as frequencies and percentages. For all statistical analyses, statistical significance was accepted at *P* < 0.05 (two-sided).

## Results

### High-Incidence Departments

**FIGURE 1:**
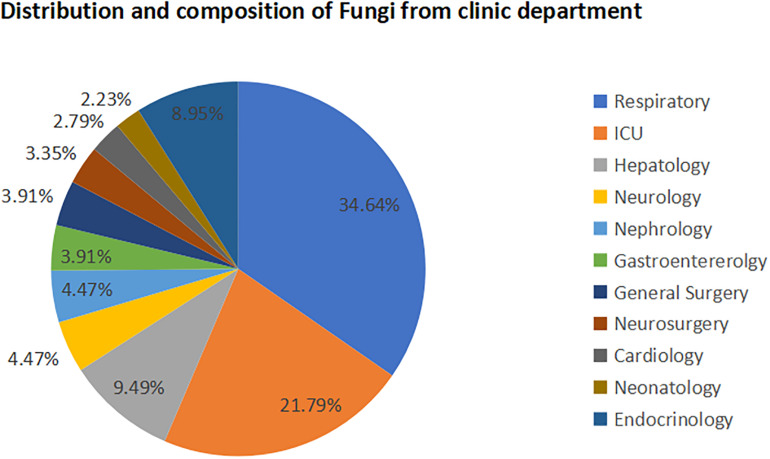
Distribution of isolated fungi strains in clinic departments. A total of 179 fungal strains were isolated from clinical patients with systemic internal diseases (49 females and 130 males). Of those, 34.64% were from the respiratory department, 21.79% were from the intensive care unit, 9.50% were from the hepatology department, 8.95% were from the endocrinology department, and 4.47% were from the neurology department and nephrology department separately.

A total of 179 fungal infection patients (49 females, 130 males) were included. Of those, 34.64% patients (*n* = 62) were in the respiratory department, 21.79% patients (*n* = 39) were in the intensive care unit (ICU), and 9.50% patients (*n* = 17) were in the hepatology department (Figure 1).

### Susceptibility System Analysis

The susceptible systems for infection were the respiratory tract, urinary tract, and gastrointestinal tract. Strains of fungi were isolated from the infective tissues or secretions of these patients, with 68.72% (123/179) coming from sputum, 9.50% (17/179) from urine, and 9.50% (17/179) from feces (Table 1).

**TABLE 1:** Susceptibility system analysis of invasive fungi infection in clinical patients with systemic internal diseases.

Clinical sample	*C. albicans* (*n*)	*C. krusei* (*n*)	*C. glabrata* (*n*)	*C. parapsilosis* (*n*)	*C. tropicalis* (*n*)	*Aspergillus* (*n*)	*Cryptococcus neoformans* (*n*)	Total	Percentage (%)
Sputum	89	5	5	2	6	16	0	123	68.72
Urine	12	3	0	1	1	0	0	17	9.50
Feces	13	2	1	1	0	0	0	17	9.50
Blood	1	0	3	2	0	0	0	6	3.35
Secretion retained in the tracheal catheter	6	0	0	0	0	0	0	6	3.35
Bile	4	1	0	0	0	0	0	5	2.79
Cerebrospinal fluid	0	0	0	0	0	0	1	1	0.56
Other	2	0	1	1	0	0	0	4	2.23
Total	127	11	10	7	7	16	1	179	
Total percentage (%)	70.95	6.15	5.59	3.91	3.91	8.93	0.56		100

### Analysis of Fungal Species in Clinical Infection

The invasive fungi isolated were primarily *Candida*, which accounted for 90.50%, with the remaining being *Aspergillus*, which accounted for 8.93%, and *Cryptococcus neoformans*, which accounted for 0.56%. Among the *Candida* species, *Candida albicans* accounted for 70.95%, *Candida krusei* accounted for 6.15%, *Candida glabrata* accounted for 5.59%, *Candida parapsilosis* accounted for 3.91%, and *Candida tropicalis* accounted for 3.91%.

### *In vitro* Susceptibilities Among Non-*Aspergillus* Fungi

Of the 179 invasive fungi detected, we analyzed the susceptibility of 163 strains of non-*Aspergillus* fungi to five kinds of antifungal drugs. The results showed that the drug susceptibility rate of the non-*Aspergillus* fungi for amphotericin B was 100.0%. The susceptibility rates for 5-FC and voriconazole were, respectively, 72.73 and 81.82% for *C. krusei*, 98.43 and 100% for *C. albicans*, and 100% for both drugs for *C. glabrata*, *C. parapsilosis*, and *C. tropicalis*. The susceptibility rates of fluconazole and itraconazole were, respectively, 0 and 54.55% for *C. krusei*, 20 and 20% for *C. glabrata*, and 57.14 and 57.14% for *C. tropicalis*. The resistance rate of *C. tropicalis* for both fluconazole and itraconazole was 41.43% (Table 2).

**TABLE 2:** *In vitro* susceptibility among non-*Aspergillus* fungi.

Fungal species	Number	Fluconazole			Itraconazole			Voriconazole			5-FC			Amphotericin B		
		S	I	R	S	I	R	S	I	R	S	I	R	S	I	R
*C. albicans*	127	97.64	2.36	0	97.64	1.57	0.79	100	0	0	98.43	0	1.57	100	0	0
*C. krusei*	11	0	0	100	54.55	45.45	0	81.82	0	18.18	72.73	27.27	0	100	0	0
*C. glabrata*	10	20.00	80.00	0	20.00	80.00	0	100	0	0	100	0	0	100	0	0
*C. parapsilosis*	7	100	0	0	100	0	0	100	0	0	100	0	0	100	0	0
*C. tropicalis*	7	57.14	1.43	41.43	57.14	1.43	41.43	100	0	0	100	0	0	100	0	0
*Cryptococcus neoformans*	1	0	100	0	0	100	0	100	0	0	100	0	0	100	0	0

## Discussion

Invasive fungal infections have been associated with increased morbidity and mortality, and the number of patients at risk of suffering invasive fungal infection is increasing (Ibáñez-Martínez et al., 2017). Several factors can contribute to this effect, and they include the widespread adoption of aggressive immunosuppressive therapy (e.g., chemotherapy, transplants), the use of new immune-modifying drugs among certain patient populations, and the increasing use of invasive devices such as central venous catheters (Enoch et al., 2017).

Our data have indicated that the clinical specimens of invasive fungi were mainly from a few departments, the respiratory department, the ICU, and the hepatology department, from which the composition breakdown was 34.64, 21.79, and 9.50%, respectively. We can conclude from our results that patients in the departments that presented high rates of invasive fungal infections, specifically the respiratory department, ICU, and hepatology department, should include special attention during clinical treatment. Patients in the ICU often have a serious illness, are committed to long-term stays in the hospital, and are subjected to extensive use of antibiotics, hormones, or immunosuppressants and invasive operations, all of which could increase the susceptibility of secondary fungal infection. Bassetti et al. (2017) suggested that further investigation was needed to determine the incidence of invasive aspergillosis in the ICU, its relationship with influenza outbreaks, the clinical impact of rapid diagnosis, and the significance of combination treatment.

Previous studies have revealed that fungal infections were identified primarily in the respiratory tract (Smith and Kauffman, 2012) and urinary tract. Furthermore, 86.03% of infective patients were persons over 60 years old who had weak mucociliary clearance ability and more bronchial gland hyperplasia and secretion (Anaissie et al., 2009). We found that, besides the respiratory tract and urinary tract, the gastrointestinal tract was also a susceptible system in which deep fungal infection appeared in patients with systemic diseases.

In this study, 179 strains of invasive fungi were identified and detected. *Candida* was the main pathogen causing invasive fungal infection in patients with systemic internal diseases. Among the *Candida* species infecting those patients, *Candida albicans* was the most frequently recognized, followed by *C. krusei*, *C. glabrata*, *C. parapsilosis*, and *C. tropicalis*. The rate of *C. glabrata* infection increased when comparing the patients with sepsis (Bloos et al., 2013).

The antifungal antibiotics were categorized into different groups including azoles, polyenes, fluoropyrimidine analogs, echinocandins, morpholines, allylamines, thiocarbamates, and 5-FC. In this study, we analyzed the sensitivity of 163 strains of non-*Aspergillus* fungi to five antifungal drugs commonly used in clinics. The results of the drug sensitivity testing showed that all of the invasive fungi in this study were 100% sensitive to amphotericin B, and over 70% of invasive fungi were sensitive for 5-FC (only 1.57% *C. albicans* with drug resistance). This suggested that amphotericin B and 5-FC should be considered more in clinical treatment (Ramana et al., 2013).

Enoch et al. declared that the epidemiology of *Candida* infections has changed in the last decade, with a gradual shift from *C. albicans* to non-albicans candida strains that may be less susceptible to azoles (Enoch et al., 2017). The sensitivity of the invasive fungi in this clinical study showed that there was a great difference in sensitivity to azoles. The resistance rate of *C. tropicalis* for both fluconazole and itraconazole was 41.43%. Moreover, *C. albicans* and *C. parapsilosis* were relatively sensitive to azoles. When compared with *C. albicans* and *C. parapsilosis*, *C. krusei*, *C. glabrata*, and *C. tropicalis* were less susceptible to fluconazole and itraconazole. The resistance rate of *C. krusei* for fluconazole was 100%, due to natural resistance. These data have high potential to be useful for the selection of antifungal drugs in the clinical setting.

Combination therapy with amphotericin B and azoles was recommended in cases of localized infection such as meningitis, osteomyelitis, and intra-abdominal infections (Ostrosky-Zeichner, 2008), but our data found that the susceptibility of invasive fungi to zolium drugs presented a genus difference. The frequent use of fluconazole and itraconazole in treating invasive fungi might be the cause of the increase in drug resistance. Badiee and Hashemizadeh (2014) also suggested that monitoring of drug dose was necessary to ensure that therapeutic levels are achieved for optimal clinical efficacy in order to prevent opportunistic invasive fungal infections in sensitive patients. Considering the safety of patients with systemic diseases, a systematic combination of antifungal drugs might increase the incidence of adverse reactions and complications.

There are several limitations to the study. The study population consisted of patients in South China and may not be representative of patients in the general population. Investigation of multiple fungal infections in patients with severe systemic diseases should also be a concern. Although this is a regional study, we hope that the data could further help multi-regional and worldwide epidemiological surveys, which could provide more favorable clinical evidence for the prevention and treatment of invasive fungal infection in patients with systemic internal diseases in the future.

In this study, we found that patients in the respiratory department, ICU, and hepatology department presented high rates of invasive fungal infections and should include special attention during clinical treatment. The respiratory tract, urinary tract, and gastrointestinal tract were the susceptible systems. *Candida*, especially *C. albicans*, was the main pathogen. From the perspective of drug sensitivity, amphotericin B should be given priority in treating the non-*Aspergillus* fungi infection in patients with systemic internal diseases, while the susceptibility of cynical strains to azoles was variant. These data might provide clinical evidence for the prevention and treatment of invasive fungal infection in patients with systemic internal diseases.

## Data Availability Statement

The raw data supporting the conclusions of this article will be made available by the authors, without undue reservation.

## Ethics Statement

The studies involving human participants were reviewed and approved by the Clinical Research Ethics Committee and Animal Care and Use Committee (IACUC) of The Third Affiliated Hospital of Sun Yat-sen University–Yuedong Hospital. Written informed consent to participate in this study was provided by the participants’ legal guardian/next of kin.

## Author Contributions

All authors listed have made a substantial, direct and intellectual contribution to the work, and approved it for publication.

## Conflict of Interest

The authors declare that the research was conducted in the absence of any commercial or financial relationships that could be construed as a potential conflict of interest.
